# Marker based enrichment of provitamin A content in two tropical maize synthetics

**DOI:** 10.1038/s41598-021-94586-7

**Published:** 2021-07-22

**Authors:** Dejene Kebede, Wende Mengesha, Abebe Menkir, Ayodeji Abe, Ana Luisa Garcia-Oliveira, Melaku Gedil

**Affiliations:** 1 International Institute of Tropical Agriculture, PMB 5320 Oyo Road, Ibadan, Nigeria; 2 Department of Plant Breeding and Biotechnology, Collage of Agricultural and Environmental Sciences, Makerere University, Kampala, Uganda; 3 Department of Agronomy, University of Ibadan, Ibadan, Nigeria; 4 International Maize and Wheat Improvement Center (CIMMYT), ICRAF House, UN Avenue, PO Box, Nairobi, 1041-00621 Kenya

**Keywords:** Biotechnology, Molecular biology, Plant sciences

## Abstract

Most of the maize (*Zea mays* L.) varieties in developing countries have low content of micronutrients including vitamin A. As a result, people who are largely dependent on cereal-based diets suffer from health challenges due to micronutrient deficiencies. Marker assisted recurrent selection (MARS), which increases the frequency of favorable alleles with advances in selection cycle, could be used to enhance the provitamin A (PVA) content of maize. This study was carried out to determine changes in levels of PVA carotenoids and genetic diversity in two maize synthetics that were subjected to two cycles of MARS. The two populations, known as HGA and HGB, and their advanced selection cycles (C1 and C2) were evaluated at Ibadan in Nigeria. Selection increased the concentrations of β-carotene, PVA and total carotenoids across cycles in HGA, while in HGB only α-carotene increased with advances in selection cycle. β-cryptoxanthine increased at C1 but decreased at C2 in HGB. The levels of β-carotene, PVA, and total carotenoids increased by 40%, 30% and 36% respectively, in HGA after two cycles of selection. α-carotene and β-cryptoxanthine content improved by 20% and 5%, respectively after two cycles of selection in HGB. MARS caused changes in genetic diversity over selection cycles. Number of effective alleles and observed heterozygosity decreased with selection cycles, while expected heterozygosity increased at C1 and decreased at C2 in HGA. In HGB, number of effective alleles, observed and expected heterozygosity increased at C1 and decreased at C2. In both populations, fixation index increased after two cycle of selections. The greatest part of the genetic variability resides within the population accounting for 86% of the total genetic variance. In general, MARS effectively improved PVA carotenoid content. However, genetic diversity in the two synthetics declined after two cycles of selection.

## Introduction

Maize *(Zea mays* L.*)* is a major crop for millions of people which depend on it as a prime source of food in sub-Saharan Africa^1,2^. The projected annual maize demand in SSA had been estimated at 500 million tons by the year 2020, which will surpass the demand for both wheat and rice^3^. It is also regarded as a vital crop for global nutrition^1,2^. However, most of the maize varieties grown in these parts of the region are white and they are all devoid of nutritionally important micronutrients such as pro-vitamin A (PVA), zinc, iodine and iron^4^. As a result, the people who depend on maize as a major food source suffer from diseases due to micronutrient deficiencies^5^. Micronutrient deficiencies, mainly caused by lack of vitamin A, are major health problems globally, with high severity in the developing world such as sub-Saharan Africa and South America^6^. Even though the required amount of micronutrients are small, their deficiencies have a wide range of negative health impacts which could eventually result in death^7^. According to^8^ 33 to 50% of the world population is estimated to be affected by malnutrition mainly caused by the deficiency of provitamin A.

Vitamin A deficiency (VAD) results from low level of PVA content of major staple food crops such as maize. As reported by^9^ more than 250 million children are globally affected by VAD. VAD is responsible for several disorders that include impaired iron mobilization, growth retardation, blindness and depressed immune response, increased susceptibility to infectious disease and childhood mortality and morbidity affecting 190 million preschool-age children and 19 million pregnant women in Africa and South Asia^10,11^ . Moreover, the increased susceptibility to several major diseases, such as anemia, diarrhea, measles, malaria and respiratory infections, which accounts for about 70% of childhood deaths throughout the world, is the most disastrous effects of VAD^12,13^.

Different strategies have been employed to alleviate VAD including food supplementation, food fortification, dietary diversification and staple crop bio-fortification^6^. Supplementation and food fortification are expensive, unsustainable and not amenable to implementation in most rural communities. However, bio-fortification through breeding, to improve the nutritional content of staple crops is an effective and sustainable method whereby farmers can easily access PVA foods by growing improved varieties, as nutritionally improved varieties are the long-term solutions to the problem through sustainable consumption of PVA carotenoids from the crops they grow^6,29^. The dietary habit of many people in the world, particularly in sub-Saharan Africa (SSA) where it is consumed daily makes maize a major staple crop of choice for bio-fortification^15,23^.

According to^37^, maize has a substantial genetic variation for kernel carotenoids, with total carotenoid content ranging from virtually zero to 15 μg g^−1^. The dominant carotenoids in maize kernels, in order of increasing concentration are α-carotene, β-cryptoxanthine, β-carotene, zeaxanthine, and lutein^15,18^. However, most maize varieties grown and consumed throughout the world have only 0.5–1.5 μg/g of β-carotene^19^. This genetic variability makes it possible to develop maize varieties with increased level of PVA carotenoid content.

Maize improvement for PVA carotenoid through conventional breeding approach involves the use of seed color and quantification of the concentration of carotenoids using high performance or ultra-high-performance liquid chromatography (HPLC or UPLC) laboratory assays. However, selection of genotypes for PVA based on seed color may not be reliable as seed color and PVA carotenoid contents are not always positively correlated with PVA concentration^20^. Furthermore, the cost of carotenoids quantification by HPLC or UPLC assays is too expensive to integrate in routine inbred line development or population improvement^4,14^. Marker-assisted selection (MAS) using DNA markers to select traits of interest could be used to complement conventional approaches. Markers that are tightly linked to target locus or loci are used for the identification of the causal loci which is an important pre-requisite to enable MAS in breeding programs. Genes or loci associated with PVA carotenoids have been identified and markers that aid in the selection of this trait have been developed and validated^4,20,21^. Therefore the use of these DNA markers in MAS has led to accelerated improvement of maize genotypes for their PVA contents^19^. The use of MAS can also shorten the time required for evaluation of breeding materials, improve the efficiency of selection and reduce the cost of screening and development of synthetics.

The use of MARS to develop synthetics with better yield potential and good nutritional quality is an important step in maize breeding. Synthetics can be released as variety per se besides serving as a source of better performing inbred parents^22^. These synthetics were synthesized from provitamin A rich elite inbred lines adapted to low-land tropical environments and are tolerant to major constraints in their target environments. Development of advanced inbred lines for the formation of provitamin A rich maize hybrids with better agronomic performance through recurrent selection has a paramount importance in improving the food and nutritional security of the target population.

Recurrent selection is a breeding procedure involving cycles of hybridization, selection and recombination and used to improve breeding populations and inbred lines for combining ability in the hybrid breeding program. MARS is one of the commonly used methods to increase the frequency of favorable alleles in subsequent selection cycles while maintaining genetic diversity^23^. Reported increased β-cryptoxanthine, β-carotene and zeaxanthine contents after three cycles of S1 recurrent selection. The study also indicated that this selection method was effective in improving the PVA content of different maize populations. Rapid cycling recurrent selection has also been employed to improve the total carotenoid content (TTC) as well as β-carotene in cassava^24^. Two cycles of marker assisted recurrent selection have been carried out to improve pro-vitamin A content in two maize synthetics. The present study was, therefore, conducted to determine the genetic gain in pro-vitamin A (PVA) content and examine changes in genetic diversity in two synthetics after two cycles of selection.

## Materials and methods

### Marker assisted enrichment of PVA

Two maize synthetics (HGA and HGB) were subjected to recurrent selection to improve their provitamin A content. The synthetics were developed from a cross of eight provitamin A rich inbred lines belonging to each heterotic group (HGA or HGB). There was no allelic information for the eight inbred lines constituting the synthetics. However, the inbred lines were subjected to phenotypic selection based on HPLC information regarding their carotenoid content, and inbred lines with > 15 µg g^−1^ were used to constitute the synthetics. Moreover, visual selection based on kernel color (deep orange kernel) were also used to select the inbred lines. The recurrent selection was carried out in IITA for two cycles. The plant selection was carried out using PVA molecular markers of lycE and crtRB1 genes and complemented with the phenotypic performance of plants. Sixty rows were planted from each population in 5 m long rows spaced 0.75 m apart and 0.25 m within a row. Two hundred and eighty-eight plants were tagged and plants that had at least two favorable alleles of the PVA markers and desirable ears were selected from each of HGA and HGB. The selected 50 and 52 plants were recombined to form the first cycle (C1) of HGA and HGB. To form the C2, 60 rows of plants from C1 of each HGA and HGB were planted. Two hundred and eighty-eight (288) plants were tagged and plants that had at least two favorable alleles of the PVA markers with good agronomic performances were selected from HGA and HGB, respectively. The selected 151 and 126 plants were recombined to form the C2 of HGA and HGB. Pollination was done by using bulk pollen from male rows to pollinate the female rows in both cases. Different numbers of plants and ears with favorable allels were selected and recombined in each cycle for both synthetics (Table 1). The genetic materials used in the present study involved neither wild species nor landrace collections received from gene banks. Consequently, permission to collect samples for carotenoid analysis was not required from gene banks. All experiments were conducted in line with relevant international, national and institutional guidelines and legislation.

**Table 1:** Number of plants (with favorable allels) and ears selected for each synthetic per cycle.

	Total number of plants selfed and genotyped in each cycle	Selected plants carrying favorable alleles		Number of ears selected and recombined	
		C1	C2	C1	C2
HGA	288	60	193	50	151
HGB	288	197	165	52	126

### Experimental design and field layout

An experiment consisting six rows of each MARS cycle of the two synthetic of different selection cycles were planted at IITA, Ibadan. The experiment was arranged in randomized complete block design (RCBD) with three replications and was planted in 5 m long rows with 0.75 m space between rows and 0.25 m within a row. NPK15:15:15 fertilizer was applied at the rate of 60 kg N ha^−1^, 60 kg P ha^−1^, and 60 kg K ha^−1^, at planting and an additional 60 kg N ha^−1^ was applied four weeks later. Manual weeding was done to keep the experiments weed free. The middle four rows were self-pollinated, and seed samples were collected for carotenoids analysis from self-pollinated plants.

### Analysis of carotenoids

The extraction protocol and carotenoid analysis used was the method described in^25^. Briefly, 0.5 g finely ground sample of each entry was transferred into a 50 ml glass centrifuge tube to which 6 ml of Ethanol plus 0.1% butylated hydroxyl toluene were added, vortexed for 15 s, and incubated in 85 °C water bath for 5 min. 500 μl of 80% potassium hydroxide (w/v) was added to each sample, vortexed for 15 s, and incubated in 85 °C water bath for 10 min with vortexing at about 5 min interval. Samples were then immediately placed on ice and 3 ml ice cold deionized water added to each of them, vortexed for 15 s, and 200 μl internal standard β-Apo-8′-carotenal and 4 ml hexane added. After vortexing and centrifugation, the top hexane layer formed was transferred into a new test tube. The hexane extraction was repeated twice, adding 3 ml hexane each time. Samples were allowed to dry down completely under nitrogen gas using a Turbovap LV concentrator (Caliper Life Sciences) and reconstituted in 500 μl of 50:50 Methanol: Dichloroethane.

Fifty micro-liter aliquots of each extract were injected into an HPLC system (Water Corporation, Milford, MA). The Water’s HPLC components was operated with Empower 1 software and included a 717 Plus auto sampler with temperature control set at 5 °C, Waters 1525 binary HPLC pump, and a 2996 photodiode array detector for carotenoid quantification. Carotenoids were separated by C30 Carotenoid Column (4.6 × 250 mm; 3 μm) eluted by a mobile phase gradient from 100% methanol/water (92:8 v/v) with 10 mM ammonium acetate to 50% methyl tertiary butyl ether. The flow rate was 1.0 mL/min and the solvents were HPLC grade. To maximize detection of carotenoids, absorbance was measured at 450 nm. Alpha-carotene, β-carotene (cis and trans isomers), β-cryptoxanthin, lutein, and zeaxanthin were quantified. Total carotenoid was calculated as the sum of concentrations of α-carotene, lutein, β-carotene, β-cryptoxanthine and zeaxanthine. Provitamin A was calculated as the sum of β-carotene and half of each of β-cryptoxanthin and α-carotene concentrations, since the latter two contribute 50% of the value of β-carotene as provitamin A^20^. Other derived carotenoid traits were also calculated as indicated in^20,21^, namely the ratio of carotenoids in β to α branch of the carotenoid pathway, the ratio of β-carotene to β-cryptoxanthine and the ratio of β-carotene to total carotenoids. The natural logarithms of the ratios were calculated to allow statistical analysis of the data, as the ratios followed a highly non-normal distribution. All concentrations were described in μg g^−1^ dry weight (DW).

### DNA extraction and genotyping

In cycle one, 288 individual plants were genotyped from HGA with markers crtRB1-5ʹ TE, LycE-3ʹ Indel, LycE-SNP(216), whereas 197 individual plants from HGB were genotyped with markers crtRB1-3ʹ TE, LycE-3ʹ Indel, LycE-SNP(216) as described in similar studies^26^. Previous studies have confirmed that crtRB1-3ʹ TE and crtRB1-5ʹ TE are in LD^26^. In cycle 2, the same genotyping method was used but with only two markers from each gene—crtRB1-3ʹ TE and LycE-SNP(216). For KASP assay, DNA was extracted, and the quality of DNA checked by running 2 µl of the diluted DNA sample on 1% agarose gel and quantified using a Nanodrop spectrophotometer (Thermo Fisher Scientific Inc., Denver, CO, USA). Genomic DNA samples were lyophilized to dry powder and sent to LGC genomics (UK) for single nucleotide polymorphism (SNP) genotyping using KASP assay platform.

### Deployment of high throughput SNP genotyping assay

CrtRB1-KASP specific PVA SNP markers developed by CIMMYT were used to evaluate the improvement of PVA across the different cycles for HGA and HGB populations. These markers fall on chromosome 10 of the crtRB1 gene, which regulates the hydroxylation of β-carotene to β-cryptoxanthine. Studies indicated that, this region is responsible for higher β-carotene accumulation in maize. Selection for the favorable SNPs of these markers are thought to enhance β-carotene and PVA content. Hence, the markers were employed for genotyping of the two synthetic populations. KASP assay genotyping platform was used for the genotyping. Alignment blast of all 7 SNPs to the crtRB1 gene nucleotide sequence showed that only SNP ZM0015 is located on the gene. The rest were either upstream or downstream of the crtRB1 gene.

### Assessment of genetic diversity after two cycles of selection

In order to examine the changes in allele frequencies caused by MARS after two cycles of selection, single nucleotide polymorphic (SNP) markers distributed across the genome were selected based on information from previous studies and SNPs routinely used as quality control (QC) set in IITA Bioscience Center. In total, 14–23 SNP markers from each chromosome were selected. KASP assay genotyping platform was employed for genotyping.

### Statistical analysis

A combined analysis of variance for PVA and carotenoids were computed with PROC GLM in SAS 9.3 using a mixed model^26^, considering cycles as fixed effects and blocks and runs as random effects. The significance of mean squares for the main and interaction effects were tested using the appropriate mean squares, obtained from the RANDOM option in SAS 9.3^26^. Repeatability values for PVA and each carotenoid were estimated using PROC MIXED procedure in SAS 9.3^26^. Mean separation was done using Least Significant Difference (LSD) at 0.05 level of probability^26^.

For each trait, cycle means were regressed as dependent variables on cycle numbers (0, 1, and 2) as independent variables using PROC Reg procedure in SAS 9.3^26^. The coefficient of linear regression (*b* value) provided an estimate of the gain per cycle, which was divided by the intercept and multiplied by 100 to obtain the percent response per cycle^27^.

GenAlEx 6.5 software^28^ was used to analyze the frequency of favorable SNP alleles in the course of recurrent selection in both populations. Out of the six crtRB1-KASP specific PVA markers used for genotyping one marker was excluded from the analysis due to high missing data. SNPs missing data greater than 20% in a population were excluded from the analysis.

Out of the 185 SNP markers used for the genetic diversity assessment, fifteen were missing. An additional three markers exhibited greater than 20% missing data and were excluded from further analysis. Genetic diversity indices such as number of effective alleles (A_e_), average observed heterozygosity (Ho), expected heterozygosity, fixation index and percent of polymorphic loci were calculated using GenAlEx 6.5^28^.

Analysis of molecular variance (AMOVA) was used to partition genetic variation among cycles and within cycles and Pairwise population Fst were analyzed using GenAlEx 6.5. Genetic distance among cycles was estimated according to^29^ method in GenAlEx 6.5. Percent of missing data for each cycle was also computed by taking the proportion of missing markers out of the total number of markers for each cycle.

### Human and animal rights

This research does not involve human/animal.

## Results

### Genetic gain for pro-vitamin A and other carotenoids

In the combined analysis of variance selection cycle was a significant source of variation for the five carotenoids, including pro-vitamin A (PVA) and total carotenoids (Table 2). Run × block interaction was significant for β-cryptoxanthine, α-carotene and total carotenoids (Table 2). Run and run × cycle interaction was not significant for all carotenoids (Table  2). Higher repeatability estimates were observed for all carotenoids including PVA and total carotenoids except for α-carotene (Table 3). The number of plants with favorable allele and ears selected in HGA was higher in C2 as compared to the previous cycle (C1). In HGB there was no consistent increase in numbers of plants selected from C1 to C2, however the number of ears selected in C2 were higher than the ones selected in C1.

**Table 2:** Mean squares from analysis of variance for pro-vitamin A and other carotenoids of HGA and HGB populations improved through marker assisted recurrent selection.

Source of variation	DF	Mean squares of carotenoids						
		Lutein (µg g^−1^)	Zeaxanthine (µg g^−1^)	β-cryptoxanthine (µg g^−1^)	a-carotene (µg g^−1^)	β-carotene (µg g^−1^)	provitamin A (µg g^−1^)	Total carotenoid (µg g^−1^)
Run	1	0.021	0.001	0.0001	0.005	0.01	0.02	0.002
Rep (Run)	4	5.9	4.2	1.6**	0.2*	9.1	9.7	92.1**
Cycle	5	196.5***	4.7***	0.6***	0.05*	56.5***	51.6***	350.7***
Run*Cycle	5	0.003	0.002	0.0004	0.009	0.06	0.083	0.12
Error	20	3.4	2.5	0.3	0.05	7.1	6.1	21.7

*, **, *** = Significant at *P* < 0.05, *P* < 0.01, and *P* < 0.001, respectively.

**Table 3:** Means and genetic gains for PVA and other carotenoids of HGA and HGB and their derived cycles improved through marker assisted recurrent selection.

Cycle	Lutein (µg g^−1^)	Zeaxanthine (µg g^−1^)	Β-cryptoxanthine (µg g^−1^)	a-carotene (µg g^−1^)	β-carotene (µg g^−1^)	PVA (µg g^−1^)	Total carotenoid (µg g^−1^)
HGAC0	11.3	12.7	4.6	1.1	13.7	16.5	43.3
HGAC1	10.5	11.0	4.7	1.0	14.0	16.8	41.2
HGAC2	23.6	11.2	3.9	1.2	18.9	21.5	58.8
HGBC0	22.3	11.7	4.2	1.0	19.3	21.9	58.4
HGBC1	13.1	13.3	4.7	1.1	12.0	14.9	44.2
HGBC2	18.8	11.4	4.4	1.2	17.6	20.4	53.4
Mean	16.6	11.9	4.4	1.1	15.9	18.7	49.9
Gain cycle^−1^ HGA	6.2	− 0.7	− 0.4	0.04	2.6	2.5	7.8
% Resp cycle^−1^ HGA	69.7	− 5.8	− 7.5	3.9	20.3	15.6	19.4
Gain cycle^−1^ HGB	− 1.7	− 0.1	0.1	0.1	− 0.9	− 0.8	-2.5
% Resp cycle^−1^ HGB	− 8.8	− 1.1	2.2	11.1	− 5	− 3.8	-4.6
Repeatability	0.99	0.58	0.64	0.10	0.90	0.91	0.96
CV	11	13	12	21	17	13	7.1
LSD(0.05)	1.9	ns	ns	ns	2.8	2.6	6.4

*Resp* response, *ns* non-significant.

The content of β-carotene, PVA and total carotenoids increased with selection cycles in HGA (Table 3). After two cycles of selection the levels of β-carotene, PVA and total carotenoids were increased by 40%, 30% and 36%, respectively in HGA. In HGB, only the content of α-carotene increased with selection cycle, while β-cryptoxanthine increased at C1 and decreased at C2 (Table 3). In HGA, the genetic gains per cycle for β –carotene, PVA and total carotenoid were 2.6 µg g^−1^, 2.5 µg g^−1^ and 7.8 µg g^−1^, respectively. The content of α-carotene and β-cryptoxanthine were improved by 20% and 5%, respectively, after two cycles of selection in HGB. The genetic gain per cycle was small (0.1 µg g^−1^) for both α-carotene and β-cryptoxanthine in HGB (Table 3). Even if the PVA and total carotenoids were not consistently increased in HGB, other important carotenoids (α-carotene and β-cryptoxanthine) showed an increase after two cycles of selection, which confirms the possibilities of enriching the two synthetics through recurrent selection.

### Changes in frequency of favorable SNP marker alleles

**Figure 1:**
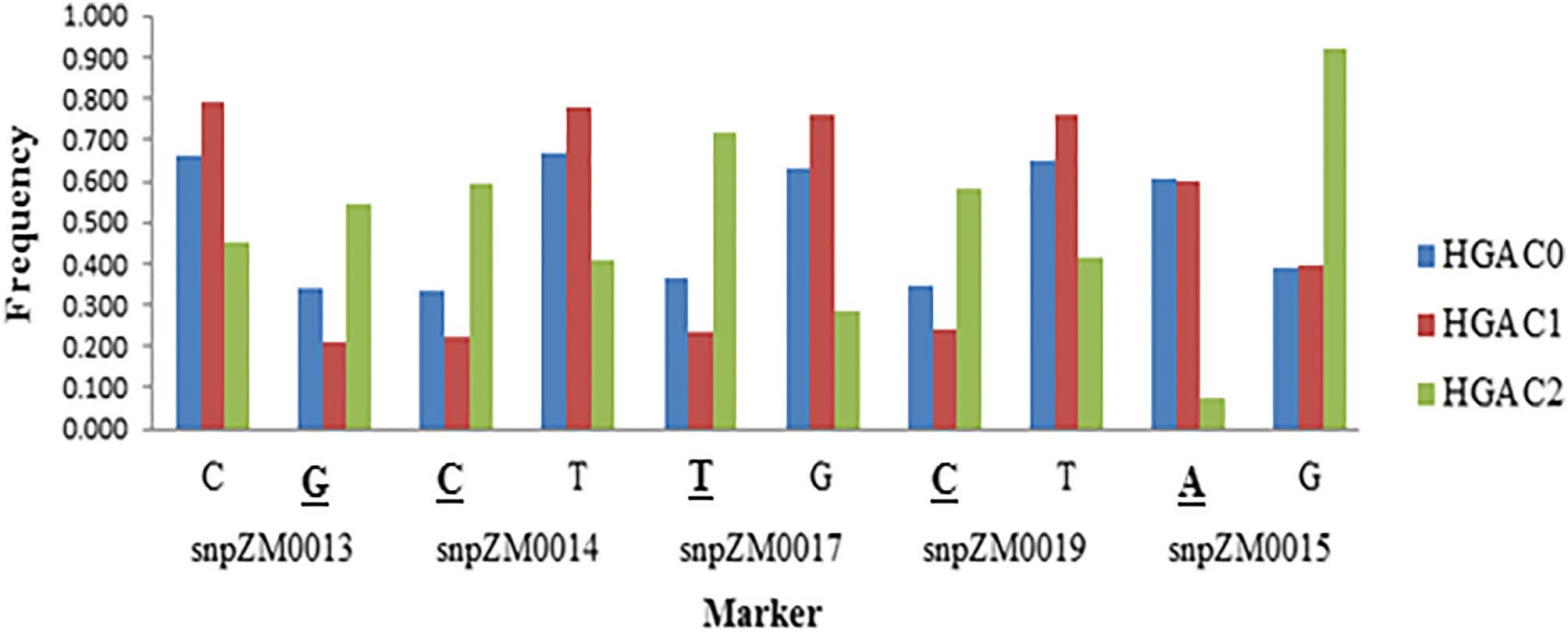
Allele frequency changes of crtRB1-KASP specific PVA marker alleles in HGA and its derived cycles. *The favorable alleles are bolded and underlined.

**Figure 2:**
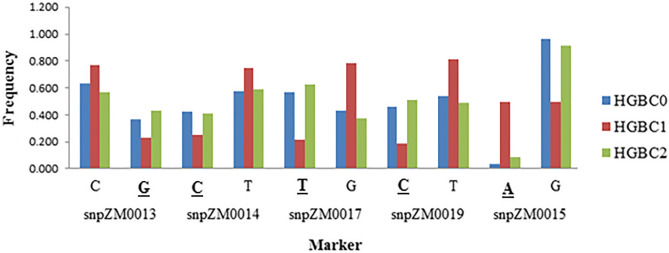
Allele frequency changes of crtRB1-KASP specific PVA marker alleles in HGB and its derived cycles. *The favorable alleles are bolded and underlined.

The mean frequency of the favorable SNPs of four crtRB1-KASP specific markers namely; snpZM0013, snpZM0014, snpZM0017 and snpZM0019, reduced at C1 and then increased at C2 in HGA (Fig. 1). In HGB, the mean frequency of the favorable alleles of three markers (snpZM0013, snpZM0017 and snpZM0019) decreased at C1 and increased at C2 (Fig. 2). However, the frequency of the favorable SNP of snpZM0015 decereased in the advanced cycle of selection (C2) in both populations. The frequency of the favorable alleles of snpZM0014 did not improve after two cycles of selection in HGB (Fig. 2). None of the marker loci got fixed in the course of selection.

### Genetic diversity study with SNP markers

In order to evaluate genetic diversity changes that occurred in the course of selection, randomly selected 60 plants from each cycle of the two synthetics were genotyped using SNP markers. Number of effective alleles and observed heterozygosity decreased with selection cycles, while expected heterozygosity increased at C1 and decreased at C2 in HGA (Table 4). On the other hand, in HGB the observed heterozygosity, number of effective alleles and expected heterozygosity slightly increased at C1 but decreased at C2 (Table 5). The percentage of polymorphic loci was slightly higher in selection cycles of HGA than those of HGB. The fixation index varied from 0.01 to 0.13 in HGA and 0.081 to 0.083 in HGB selection cycles.

**Table 4:** Genetic variability based on SNP markers computed for HGA and its selection cycles.

Cycles	# Samples ^1^	Missing data (%)^2^	% Polymorphic loci^3^	# Effective alleles^4^	Observed hetrozygosity^5^	Expected hetrozygosity^6^	Fixation Index^7^
HGAC0	60	4.6	93	1.6 ± 0.026	0.33 ± 0.015	0.32 ± 0.014	0.01 ± 0.018
HGAC1	60	3.9	94	1.6 ± 0.027	0.29 ± 0.013	0.33 ± 0.013	0.11 ± 0.019
HGAC2	60	5.5	93	1.5 ± 0.027	0.24 ± 0.013	0.27 ± 0.013	0.13 ± 0.022
Mean	60	4.7	93	1.5 ± 0.016	0.29 ± 0.008	0.31 ± 0.008	0.08 ± 0.012

^1^# Number of samples correspond to number of plants from which leaf tissue was assayed.

^2^Percent (%) of missing data is the percentage of markers that did not return data computed per cycle.

^3^Percent of polymorphic loci is the percentage of polymorphic loci to the total number of loci (polymorphic and monomorphic) per cycle.

^4^Number of effective alleles are the number of alleles that can be present in the population.

^5^Observed heterozygosity is the number of markers within a population that are heterozygous calculated per cycle based.

^6^Expected heterozygosity is the probability that at a single locus, any two alleles chosen at random from the population are different to each other.

^7^Fixation index is the measure of reduction in heterozygosity.

**Table 5:** Genetic variability based on SNP markers computed for HGB and its selection cycles.

Cycles	# Samples^1^	Missing data (%)^2^	% Polymorphic loci^3^	# Effective alleles^4^	Observed hetrozygosity^5^	Expected hetrozygosity^6^	Fixation Index^7^
HGBC0	60	3.7	88.6	1.5 ± 0.027	0.25 ± 0.015	0.27 ± 0.014	0.081 ± 0.02
HGBC1	60	6.7	93.4	1.6 ± 0.027	0.3 ± 0.014	0.32 ± 0.013	0.054 ± 0.01
HGBC2	60	5.8	94.6	1.5 ± 0.026	0.26 ± 0.014	0.29 ± 0.013	0.083 ± 0.021
Mean	60	5.4	92.2	1.5 ± 0.016	0.27 ± 0.008	0.29 ± 0.008	0.07 ± 0.011

^1^# Number of samples correspond to number of plants from which leaf tissue was assayed.

^2^Percent (%) of missing data is the percentage of markers that did not return data computed per cycle.

^3^Percent of polymorphic loci is the percentage of polymorphic loci to the total number of loci (polymorphic and monomorphic) per cycle.

^4^Number of effective alleles are the number of alleles that can be present in the population.

^5^Observed heterozygosity is the number of markers within a population that are heterozygous calculated per cycle based.

^6^Expected heterozygosity is the probability that at a single locus, any two alleles chosen at random from the population are different to each other.

^7^Fixation index is the measure of reduction in heterozygosity.

### Carotenoid concentrations of early generation lines derived from HGA and HGB

**Figure 3:**
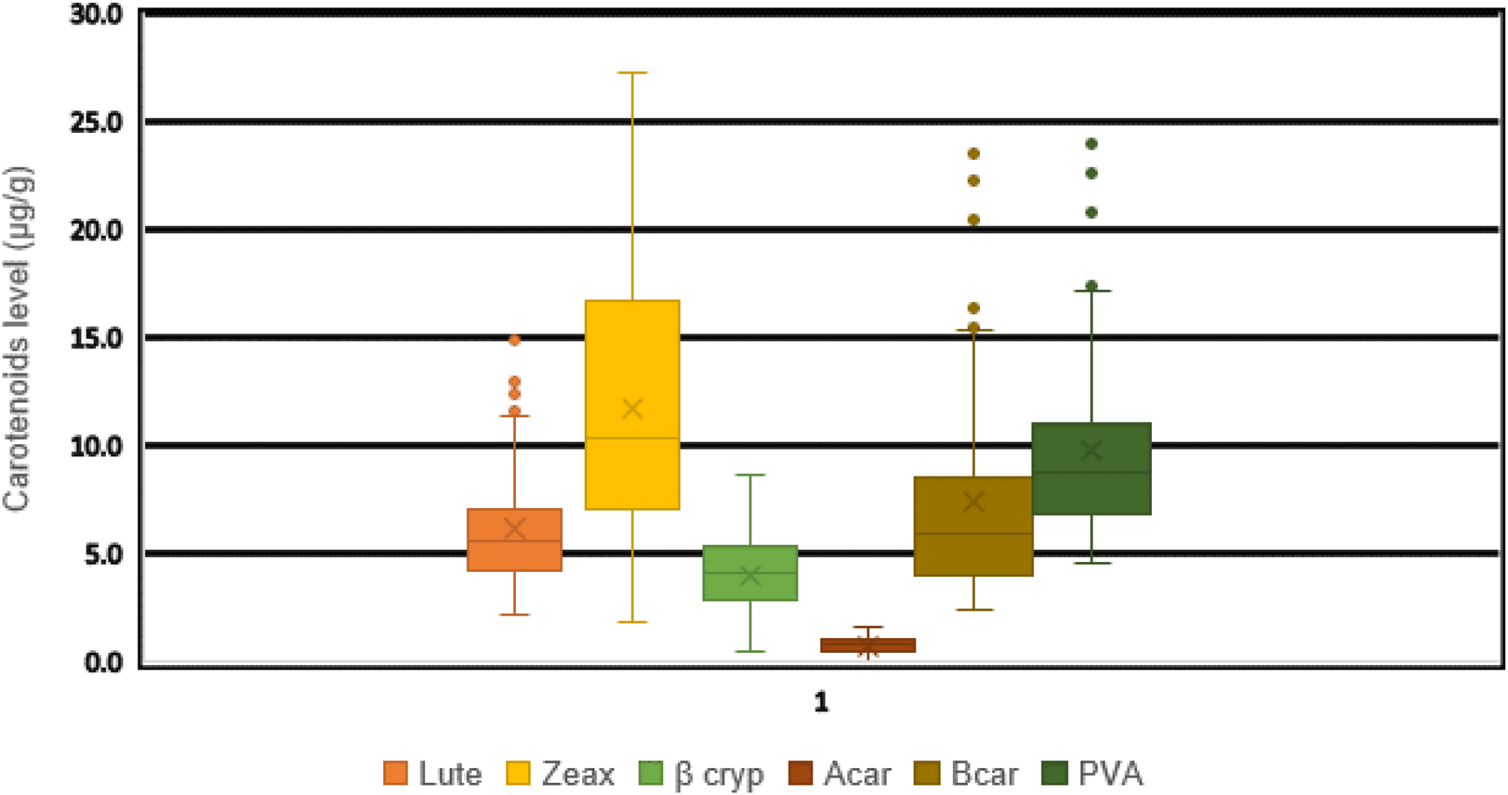
Distribution of the different carotenoids across the S4 inbred lines derived from HGA.

**Figure 4:**
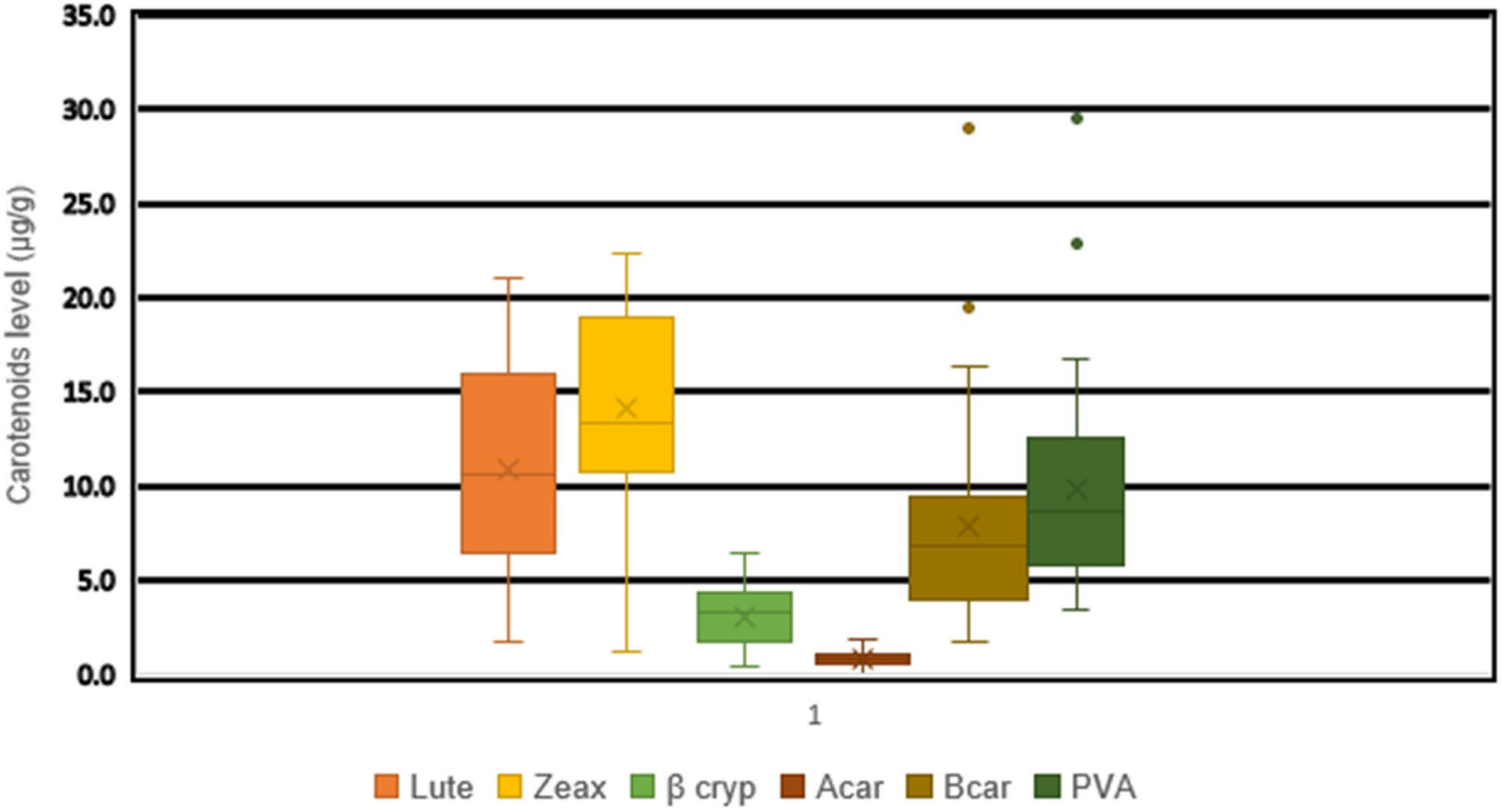
Distribution of the different carotenoids across the S4 inbred lines derived from HGB.

The result of analyses showed the presence of considerable variability among the inbred lines for important carotenoids considered in this study (Figs. 3 and 4). The genetic variability among the S4 lines derived form HGA was broader for Zeaxanthin and B-carotene. In HGB the genetic differences among inbred lines was higher for lutein and zeaxanthin (Figs. 3 and 4). The differences among S4 lines was small for α-carotene content in the two synthetics. Inbred lines derived from the two synthetics (HGA and HGB) displayed considerable variability in provitamin A content. Thirty-one S4 lines in HGA and 13 S4 lines in HGB had provitamin A contents exceeding 10 µg/g. Fourteen inbred lines in HGA and 4 in HGB had provitamin A contents of more than 15 µg/g, which is above the target level set under HarvestPlus Global Challenge Program (Figs. 3 and 4).

## Discussion

The present study was conducted to determine the genetic gain for pro-vitamin A (PVA) content and examine the extent of change in genetic diversity in two synthetics. The two synthetics were formed from pro-vitamin A rich maize inbred lines belonging to different heterotic groups developed at IITA and they were improved for pro-vitamin A content through two cycles of MARS. The observed significant difference between selection cycles for all carotenoids as well as PVA and total carotenoids suggest that selection induced significant changes in carotenoid accumulations across selection cycles, consistent with the results reported earlier^23^. The higher repeatability estimates for content of all carotenoids as well as PVA and total carotenoids indicate that these nutrients were accurately measured. This result was consistent with that reported by^30^ who observed higher repeatability estimates of carotenoids concentrations for major carotenoids including PVA and total carotenoids in maize.

Linear increase was observed for β-carotene, PVA and total carotenoids with selection in HGA, consistent with the results reported by^23,24^ also demonstrated significant improvement for β-carotene and total carotenoids with selection cycles in cassava in rapid cycling recurrent selection program. The content of PVA and other carotenoids were higher in the base population but decreased with advances in selection cycle in HGB. The possible explanation for this could be loss of plants with favorable alleles during the process of selection for desirable ears. Since a combination of molecular and phenotypic selection of ears for bright yellow to orange color and semi flint to flint kernel texture were employed for selection of plants for recombination, some plants with favorable PVA alleles could have been lost. ^20^ observed that selection of plants for PVA content based on seed color may not be reliable as seed color and PVA carotenoid contents are not always positively correlated.

^31^reported the existence of duplicated beta-carotene hydroxylase genes elsewhere in the maize genome, suggests a complex regulatory system for maize carotenoid biosynthesis, with each hydroxylase gene playing functionally different roles. Such scenario is the most probable case in our study, where there was steady increase in provitamin A content in HGA, but showed fluctuation in case of HGB. Likewise, the observed reduction in PVA content at an advanced cycle (C2) of HGB may also be due to the linkage drag from the provitamin A donor parents or loss of diversity associated to intensive selection on the synthetics^31^. The other potential causes of inconsistency could also arise from random changes in allele frequencies or genetic drift, which can cause changes in population means, particularly for a relatively small sized synthetics typical of our recurrent selection experiments^32^. Larger population size would be the correct strategy to overcome the problem of segregation distortion effects on selection in the breeding program for high provitamin A level using markers. However, our result still confirms the possibilities of developing products with higher carotenoid contents through the deployment of recurrent selection using relatively narrow based synthetics as a base population.

The current study is in line with^31^ who reported the results of 26 tropical maize populations, demonstrating the effectiveness of marker-assisted selection (MAS) for alleles of crtRB1, which is increasingly being implemented by breeding programs involved in the development of varieties with enhanced levels of provitamin A and other essential carotenoids. In this study, β-carotene was more abundant than other PVA carotenoids and increased consistently with selection cycles in HGA. After two cycles of MARS, the β-cryptoxanthine content did not increase in HGA and its improvement was very small in HGB. Similarly, the content of zeaxanthin did not increase after two cycles of selection in both populations. The result of this study suggests that hydroxylation reducing alleles of crtRB1 were selected in the process of the MARS population development.

Recently, CIMMYT has developed crtRB1 KASP specific SNP markers which are being used to screen and evaluate maize breeding populations for PVA due to their cost effectivness and shortening the time required for genotyping. Selection for the favorable SNPs of these markers enables the selections of favorable alleles of crtRB1 (5′TE, InDel4 and 3′TE) that could double the β-carotene content in maize endosperm (Yan et al., 2010). In this study, the frequency of the crtRB1 KASP SNP markers associated with favourable alleles for PVA increased after two cycles of MARS. Out of the six crtRB1 KASP markers used for genotyping, the frequency of favorable alleles of four and three markers increased after two cycle of selection in HGA and HGB, respectively. The gradual increase in the frequency of favorable alleles was in line with the findings of^27,33^ who observed that MARS increased the frequencies of the favorable allele.

After two cycles of MARS, the PVA content of HGA was 21.5 µg g^−1^, which is higher than the 17.3 µg g^−1^ previously reported by^26^ for PVA inbred lines. This result was also greater than the PVA target level of 15 µg g^−1^ set by HarvestPlus Global Challenge Program to overcome vitamin A deficiency^19^. The higher response per cycle of selection and genetic gain observed for PVA and other carotenoids suggest that marker assisted recurrent selection was effective in improving the PVA content and other carotenoids when there is adequate diversity in a population under selection^23^.

The genetic variability in a population could possibly be maintained through random mating of individuals which was the case in the present study where the observed heterozygosity and number of effective alleles slightly increased from C0 to C1 and decreased at C2 in HGB. However, there was reduction in the number of effective alleles, observed heterozygosity and expected heterozygosity after two cycle of marker assisted recurrent selection for HGA, which is consistent with other earlier works (Vogel 2010). The reason for the reduction of these genetic diversity indices could be the effect of changes in frequency of favorable and unfavorable alleles^27^. It could also be because of selection and genetic drift^4,35^. A similar result was reported by^33,36^. The average percent polymorphic loci were higher in HGA (94%) than HGB (92.2%) suggesting that HGA had higher genetic variability than HGB. Expected heterozygosity was generally greater than observed heterozygosity within selection cycles for both HGA and HGB. This might suggest that the two populations were different with different alleles being fixed at advanced selection cycles. This result was consistent with that of^35^ who reported larger expected heterozygosity than observed heterozygosity for two maize RRS selection populations.

The increase in fixation index in the course of selection suggests that homozygosity increased at the advanced selection cycle^37^. ^38^who studied the genetic diversity of two composite maize recurrent selection populations reported an increase in the proportion of homozygotes with selection cycles. The decrease in the average effective number of alleles from 1.6 at C0 to 1.5 at C2 in HGA as well as the increase in fixation index after two cycles of selection in both populations suggests that the populations are gradually moving towards allele fixation^34^. The reduction in the level of heterozygosity and increased fixation index from C0 to C2 imply loss of heterozygosity possibly because of positive assortative mating and selection of homozygotes^28^. The reduction in expected heterozygosity and the increase in the level of inbreeding resulting from response to selection were in line with the findings of^33^ who observed a decrease in level of gene diversity, increase level of inbreeding and changes in allele frequency in a bi-parental maize population improved through MARS.

Allele frequency changes allows for the identification of favorable or unfavorable alleles. ^39,40^ found a high mean frequency at C1 while a third population showed no difference in mean frequency of favorable alleles between C1 and C2. Our results suggest that breeders may conduct two cycles of MARS to develop superior inbred lines with enhanced levels of essential carotenoids including provitamin A. The combination of favorable alleles in the present study reveals that majority of the S4 inbred lines derived from both synthetics, had higher frequencies of favorable alleles for provitamin A and other important carotenoids. This identification of S4 lines with provitamin A contents of up to 29 µg/g suggested that breeding scheme was effective in enhancing genetic gain for carotenoid contents in the synthetics through recombination of alleles.

In summary, improvement in PVA and changes in genetic diversity of the two maize synthetics subjected to MARS was investigated. The PVA content increased with advances in selection cycles in HGA. MARS effected desirable changes in the frequency of favorable alleles for PVA in both populations. It was therefore, concluded that it can be used for improving PVA content of tropically adapted maize germplasm.
